# Role of Kif15 and its novel mitotic partner KBP in K-fiber dynamics and chromosome alignment

**DOI:** 10.1371/journal.pone.0174819

**Published:** 2017-04-26

**Authors:** Nathalie Brouwers, Nuria Mallol Martinez, Isabelle Vernos

**Affiliations:** 1 Cell and Developmental Biology Program, Centre for Genomic Regulation (CRG), The Barcelona Institute of Science and Technology, Dr. Aiguader 88, Barcelona, Spain; 2 Universitat Pompeu Fabra (UPF), Barcelona, Spain; 3 Institució Catalana de Recerca i Estudis Avançats (ICREA), Pg. Lluis Companys 23, Barcelona, Spain; Virginia Tech, UNITED STATES

## Abstract

Faithful segregation of the genetic material during the cell cycle is key for the continuation of life. Central to this process is the assembly of a bipolar spindle that aligns the chromosomes and segregates them to the two daughter cells. Spindle bipolarity is strongly dependent on the activity of the homotetrameric kinesin Eg5. However, another kinesin, Kif15, also provides forces needed to separate the spindle poles during prometaphase and to maintain spindle bipolarity at metaphase. Here we identify KBP as a specific interaction partner of Kif15 in mitosis. We show that KBP promotes the localization of Kif15 to the spindle equator close to the chromosomes. Both Kif15 and KBP are required for the alignment of all the chromosomes to the metaphase plate and the assembly of stable kinetochore fibers of the correct length. Taken together our data uncover a novel role for Kif15 in complex with KBP during mitosis.

## Introduction

Faithful segregation of the genetic material during cell division is key for the continuation of life. Central to this process is the formation of the bipolar spindle that moves chromosomes to first align them on the metaphase plate and then provide the forces to segregate them to the daughter cells. Molecular motors play essential roles both in bipolar spindle assembly and chromosome alignment.

In mammalian cells, the homotetrameric kinesin Eg5 plays a major role in spindle bipolarity by generating forces driving centrosome separation and by cross-linking and sliding anti-parallel microtubules (MTs) apart [1]. However, Eg5 independent mechanisms participate in the establishment and maintenance of spindle bipolarity. Indeed, bipolar spindles can form in the absence of Eg5 when dynein activity is also impaired [2,3] and in some organisms Eg5 is not required for bipolar spindle formation [1,4,5]. Moreover Eg5 is not essential for the maintenance of spindle bipolarity in metaphase [6,7].

The kinesin-12 family member Kif15 (also named Hklp2) is a plus-end directed kinesin that localizes in a mitosis-specific manner to both spindle microtubules and chromosomes [2,3,6,7]. While Kif15 is dispensable for bipolar spindle formation in normal cell division, it becomes essential when Eg5 activity is perturbed [6,7]. Moreover, Kif15 overexpression can take over all functions of Eg5 in spindle formation upon acute Eg5 inhibition [4,5,7] allowing cells to survive upon permanent Eg5 inhibition [6–8].

The mechanism by which Kif15 and Eg5 promote spindle bipolarity is most probably not redundant. Indeed, the two motors have spatially and temporally distinct localization patterns. Eg5 interacts with the centrosomal MTs before nuclear envelope breakdown (NEBD) and becomes later enriched towards the spindle poles. Instead Kif15 only associates with the spindle MTs in a TPX2 dependent manner after NEBD [6,7,9]. It associates with higher affinity with the K-fiber MTs (K-MTs) and also localizes to the mitotic chromosomes [6,7,10]. Interestingly, Eg5 overexpression in HeLa cells is compatible with bipolar spindle assembly whereas Kif15 overexpression promotes aberrant microtubule organizations [6,7], pointing to very different modes of action. While Eg5 properties and activity are well established, there are currently divergent data for Kif15 oligomerization state and mode of action. Eg5 is a homotetramer that cross-links MTs preferentially in the antiparallel orientation and slides them apart [1]. Instead Kif15 and its orthologs were either described as dimers [2,3,9,11–13], or tetramers [14,15]. Different models have been therefore proposed for its role in bipolar spindle assembly [6,7,11]. Interestingly, it was recently shown in vitro that Kif15 accumulates at microtubule plus-ends and suppresses catastrophe events [14]. Moreover, the same authors showed that it can crosslink microtubules and move them relative to one another, promoting the formation of parallel microtubule arrays. Interestingly, others studies suggested a role for Eg5 and Kif15 in chromosome movements [4,5,16]. Moreover, it was proposed that Kif15 modulates K-fiber generated forces opposing the Eg5 generated forces [6,7,10].

To gain further insights into the function of Kif15, we have searched for putative novel interaction partners. We identified KBP as a protein that interacts with its motor domain specifically during mitosis. KBP promotes Kif15 localization to the spindle equator close to the chromosomes, presumably at MT plus-ends. Using live cell microscopy we show that Kif15 and its associated partner KBP are required for K-fiber assembly and for the complete alignment of all the chromosomes to the metaphase plate.

## Results

### KBP is a novel interaction partner of Kif15

To obtain better insights into the role of Kif15 during mitosis we used a SILAC (stable isotope labeling with amino-acids in cell culture) approach combined with quantitative mass spectrometry to identify novel interaction partners. Using a stable HEK293T cell line expressing flag-tagged Kif15 we performed anti-flag pull-down experiments from cell synchronized in mitosis. Three independent flag-Kif15 pull-downs systematically retrieved KBP as the candidate partner with the highest score (S1A Fig). This protein, KIAA1279, also named Kif1-binding protein, had been previously described as interacting with members of the kinesin-3 family in interphase tissue culture cells, primary neuronal cultures and neuronal tissues [17,18]. During the course of this study it was also found in two independent studies to interact with Kif15 [19,20]. However, the function of KBP or of its interaction with Kif15 in mitosis have not been reported.

**Fig 1:**
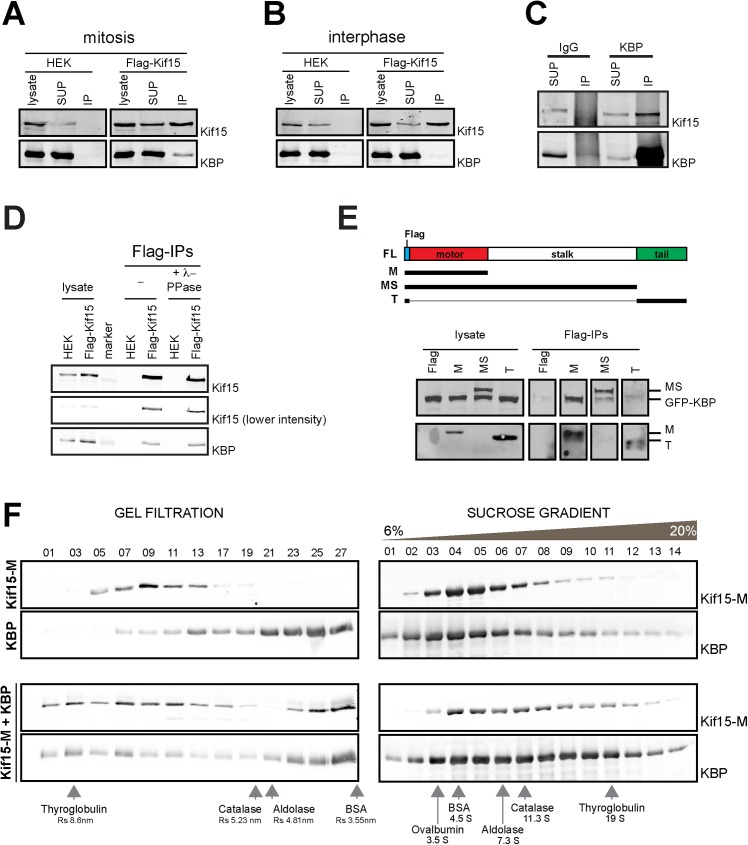
KBP is a novel interaction partner of Kif15. **(A-B)** Non-transfected or stable Flag-Kif15 expressing HEK293T cells were synchronized in mitosis **(A)** or interphase **(B)** and the corresponding cell lysates were used for anti-Flag immunoprecipitation. Cell lysates and immunoprecipitated proteins were resolved by SDS-PAGE and detected by immunoblotting with anti-Kif15 or anti-KBP antibodies. **(C)** Cell lysates of HEK293T cells synchronized in mitosis were subjected to anti-KBP immunoprecipitation. Cell lysates and immunoprecipitated proteins were resolved by SDS-PAGE and detected by immunoblotting with anti-Kif15 and anti-KBP antibodies. **(D)** Non-transfected or transiently expressing Flag-Kif15 HEK293T were synchronized in mitosis. Cell lysates were collected and incubated in the presence or absence of **λ**-phosphatase. Treated and non-treated cell lysates were subjected to Flag immunoprecipitation. Cell lysates and immunoprecipitated proteins were resolved by SDS-PAGE and detected by immunoblotting with anti-Kif15 and anti-KBP antibodies. **(E)** HEK293T cells were transiently transfected with GFP-KBP and different truncation constructs of Flag-Kif15 and synchronized in mitosis. Cell lysates were subjected to Flag immunoprecipitation. Cell lysates and immunoprecipitated proteins were resolved by SDS-PAGE and detected by immunoblotting with anti-Flag and anti-KBP antibodies. **(F)** Western blot analysis of selected fractions from gel filtration and sucrose gradient centrifugation experiments using purified recombinant Kif15-M-His, KBP-His individually or in combination as indicated on the left. Proteins were detected by immunoblotting using anti-His (Kif15-M) and anti-KBP antibodies as indicated on the right. The sucrose gradient fractions corresponding to 6 to 20% sucrose are shown.

**Fig 2:**
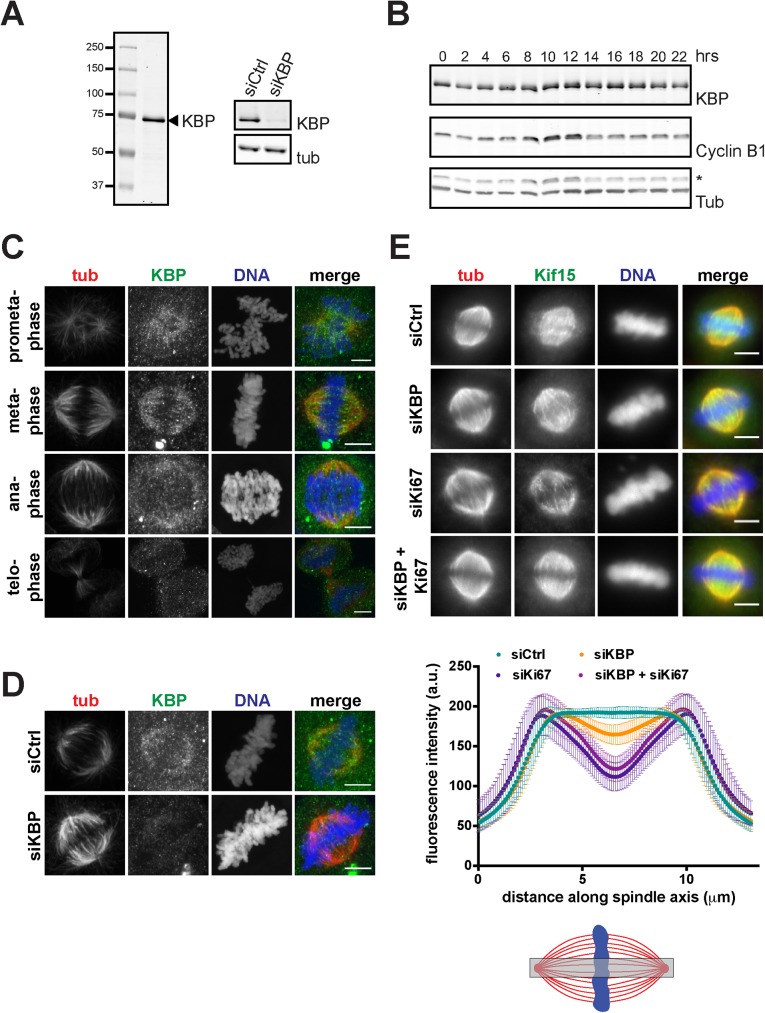
KBP promotes localization of Kif15 to the spindle midzone. **(A)** Left panel: Immunoblot of SDS-PAGE resolved HeLa cell lysate with affinity-purified anti-KBP antibody. One single band of about 72kDa is recognized. Right panel: Immunoblot of control or KBP silenced HeLa cell lysates. Membranes were probed with anti-KBP and anti-tubulin, as a loading control, antibodies. **(B)** Cell cycle regulation of KBP expression. HeLa cells were synchronized by double thymidine block and released for the indicated time periods. Cell lysates were SDS-PAGE resolved and immunoblotted with anti-KBP antibody. Anti-Cyclin B1 staining was used to monitor progression through the cell cycle and anti-tubulin staining was used as a loading control (* indicates the Cyclin B1 signal). **(C)** Immunofluorescence showing representative images of prometa-, meta-, ana- and telophase cells. Cells were pre-extracted, fixed and stained with anti-KBP and anti-tubulin antibodies (scale bar, 5 μm). **(D)** Immunofluorescence showing representative images of metaphase cells in control silenced or KBP silenced cells. Cells were pre-extracted, fixed and stained with anti-KBP and anti-tubulin antibodies (scale bar, 5 μm). **(E)** Upper panel: Immunofluorescence of control, KBP, Ki67 or KBP/Ki67-silenced cells showing the reduced Kif15 signal at the metaphase plate (scale bar 5, μm). Lower panel: Quantification of Kif15 signal in a region central in the metaphase spindles (as indicated by the grey box in the scheme). Kif15 signal was measured as intensity profiles along the spindle axis. Plotted is the average Kif15 signal with standard deviation (four independent experiments with at least 39 cells per condition, per experiment for KBP silencing, and one experiment with at least 61 cells per condition for Ki67 or KBP/Ki67 silencing).

We confirmed that Kif15 and KBP do interact by western blot analysis of flag-Kif15 pull-downs using the stable flag-Kif15 expressing HEK293T cell line (Fig 1A). Moreover we could also detect an interaction between the endogenous proteins by immunoprecipitating endogenous KBP from mitotic HEK293T cells (Fig 1C). Interestingly, although Kif15 and KBP are present during the entire cell cycle (Fig 2B and [6], the two proteins co-immunoprecipitate exclusively in mitosis (Fig 1A and 1B). Pull-down experiments in the presence of **λ**-phosphatase suggested that the interaction was not regulated by phosphorylation (Fig 1D).

To determine which region of Kif15 interacts with KBP we expressed various flag-tagged truncated Kif15 fragments by transient transfection in HEK293T cells synchronized in mitosis. Western blot analysis of the anti-flag pull-downs showed that GFP-KBP (Fig 1E) and endogenous KBP (data not shown) interact with the motor domain of Kif15.

To determine whether the interaction between the motor domain of Kif15 and KBP is direct, we performed gel filtration and sucrose gradient centrifugation experiments using a recombinant His-tagged Kif15 fragment corresponding to its motor domain and part of the stalk sufficient for dimerization (Kif15-M-His, amino acids 1–714) and full-length His-tagged KBP. The distribution of the proteins when incubated alone or in combination changed both in the sucrose gradients and in gel filtration, indicating that the two proteins interact directly (Fig 1F) in an ATP-independent manner (S1B Fig). The calculations of the native molecular weights using their sedimentation coefficient and Stokes radius for the individual proteins indicated that KBP is a monomer whereas Kif15-M-His is a dimer. Interestingly, the calculated native molecular weight of the complex formed by the two proteins suggested a stoichiometry of one Kif15-M dimer with two KBP proteins. These data suggested that KBP interacts directly with Kif15 through its motor domain forming a 2:2 complex (S1B Fig).

Altogether, our data show that KBP is a novel mitotic partner of Kif15 that interacts directly with its motor domain.

### The novel Kif15-interacting protein KBP localizes to the spindle and promotes Kif15 localization to the spindle equator

Next we looked at KBP localization and function during mitosis. Immunofluorescence analysis on HeLa cells did not reveal any specific localization for KBP during interphase (data not shown) but in mitotic cells it did localize along the spindle MTs during prometaphase and metaphase (Fig 2C and 2D). Transient expression of flag-KBP in HeLa cells confirmed this mitotic localization (S2E Fig).

We then examined whether KBP is required for Kif15 mitotic localization. Silencing of KBP efficiently reduced its levels to 12±6% (Fig 2A). Immunofluorescence analysis showed that in the absence of KBP, Kif15 did localize to the spindle MTs in prometaphase and metaphase (Fig 2E and S2A Fig) but it seemed to be reduced at the spindle equator and chromosomes (Fig 2E and S2A Fig). To examine this change of localization we measured the intensity of the Kif15 signal along the pole-to-pole spindle axis in control and KBP-silenced cells. We found that indeed Kif15 signal was reduced by ~12% compared to controls at the equator of the spindle and chromosomal area (Fig 2E). Although the reduction was relatively small it was consistent and significant in four independent experiments. However, it was not as pronounced as in cells silenced for Ki67, a chromosome associated protein previously shown to interact with Kif15 (Fig 2E) [6]. Altogether our results show that KBP promotes Kif15 localization to the spindle equator and the chromosome area.

### KBP is not required for bipolar spindle assembly but contributes to Kif15-dependent chromosome alignment

We then addressed the role of KBP during mitosis by immunofluorescence analysis on fixed cells. We did not find any significant difference on the proportion of bipolar and monopolar spindles in control and KBP-silenced cells (data not shown). Moreover, there were no differences either in the spindle length (S2B Fig). Since the dominant role of Eg5 in bipolar spindle establishment masks in large part the contribution of Kif15 in this process [6–8], we examined the role of KBP in cells that grow in the absence of Eg5 activity. HeLa cells were selected under constant inhibition of Eg5 as previously described [8]. These cells grew and divided independently of Eg5 activity (Eg5 independent cells, EICs) but required Kif15 activity to form a bipolar spindle as previously described [8]. In contrast, KBP silencing did not significantly increase the number of monopolar spindles compared to control or Kif15 silencing, indicating that it is not required for the establishment of bipolar spindles (S2C Fig). These results therefore suggest that KBP does not contribute to the function of Kif15 in promoting spindle bipolarity.

**Fig 3:**
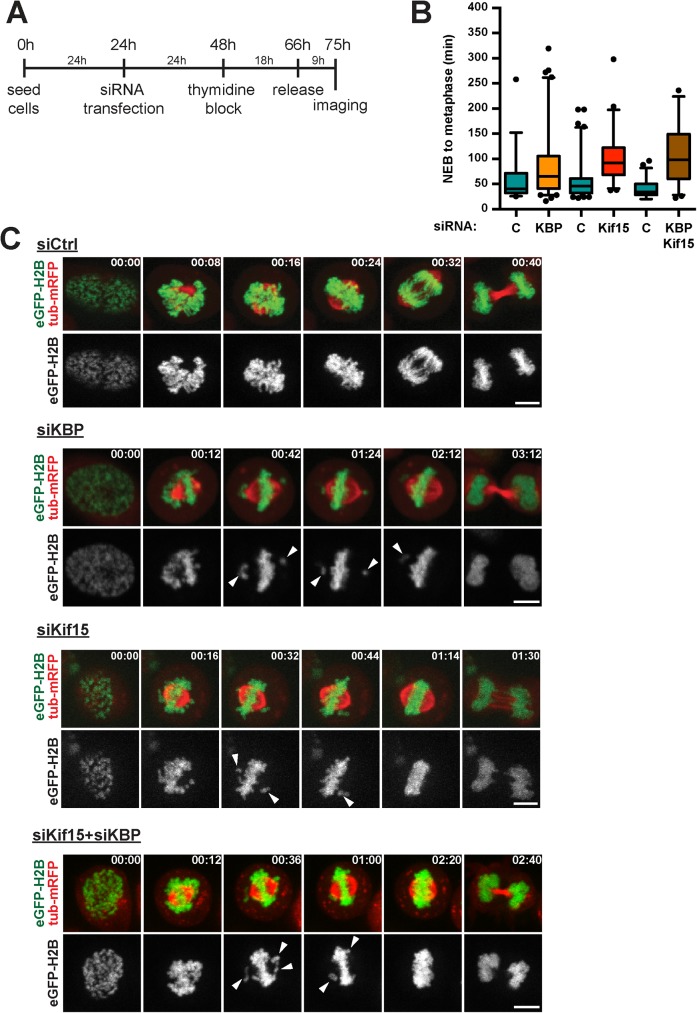
KBP and Kif15 function in efficient chromosome alignment. **(A)** Scheme outlining the experimental set-up of the live-cell imaging. **(B)** Quantification of prometaphase duration as observed in the live-cell imaging in control (C), KBP-, Kif15- or KBP/Kif15-silenced cells. Prometaphase was defined as the time needed for cells to progress from NEBD till anaphase onset. Time points are represented in box-and-whisker plots. Boxes show the upper and lower quartiles (25–75%) with a line at the median, whiskers extend from the 5^th^ to the 95^th^ percentile. Dots represent the outliers (data were compared using Mann-Whitney *U* tests, ** means *p* ≤ 0.01, **** means *p* ≤ 0.0001). **(C)** Representative stills from the live-cell imaging of control, KBP-, Kif15- or KBP/Kif15-silenced cells. Arrowheads highlight chromosomes that show less efficient alignment to the metaphase plate. Time is indicated in the upper right corner as hh:mm. Scale bars represent 5μm.

Next we addressed KBP function during mitosis by live cell imaging. We followed dividing KBP-silenced cells at high temporal and spatial resolution by time-lapse imaging. HeLa cells stably expressing eGFP-H2B and Tub-mRFP were synchronized with a single thymidine block and released 9 hours before imaging (Fig 3A). KBP-silenced cells showed a significant delay in mitosis (S3A Fig and S1 and S2 Movies) spending on average 100.6±68.3 min from NEB to the start of telophase, instead of 70.4±45.5 min for control cells (*p* = 0.0055). A more detailed analysis of the movies revealed that the KBP-silenced cells were specifically delayed during prometaphase, remaining in this phase 90.4±69.5 min on average instead of 59.4±44.9 min for control cells (*p* = 0.0046, Fig 3B and 3C). For comparison we also performed the same analysis for cells silenced for Kif15. Interestingly we observed a similar, yet slightly more pronounced, prometaphase delay as the silenced cells remained in prometaphase for 100.1±48.6 min on average instead of 55.7±37.1 min for control cells (*p*<0.0001) (Fig 3B and 3C and S3 Movie). Careful analysis of the movies revealed that the prometaphase delay could be ascribed to defects in chromosome alignment to the metaphase plate. Although chromosome congression seemed to occur normally for most chromosomes, 31% of the KBP-silenced cells had one or a few chromosomes remaining in close proximity to a spindle pole for extended periods of time (Fig 3C and S1–S3 Movies). The chromosome alignment defects was also observed in fixed KBP-silenced cells and could be rescued by exogenous expression of recombinant KBP (S2D Fig).

The analysis of the movies of Kif15-silenced cells undergoing mitosis showed that 44% of the cells had similar chromosome alignment defects as the KBP-silenced cells (Fig 3C and S1–S3 Movies). These results suggested that the two proteins are required for chromosome alignment. To determine whether they may function through the same pathway, we then monitored mitosis by live imaging of cells co-silenced for Kif15 and KBP. The co-silenced cells showed phenotypes that were very similar to those of cells silenced for Kif15 or KBP alone. Indeed they had an extended prometaphase lasting 106.4±61.5 min instead of 39.8±17.2 min in control cells (*p*<0.0001) and 40% of the co-silenced cells showed defects in chromosome alignment. These results suggested that Kif15 and its mitotic partner KBP promote chromosome alignment through the same pathway (Fig 3B and 3C and S4 Movie).

### Kif15 localization to the chromosomes and spindle equator is required for chromosome alignment

**Fig 4:**
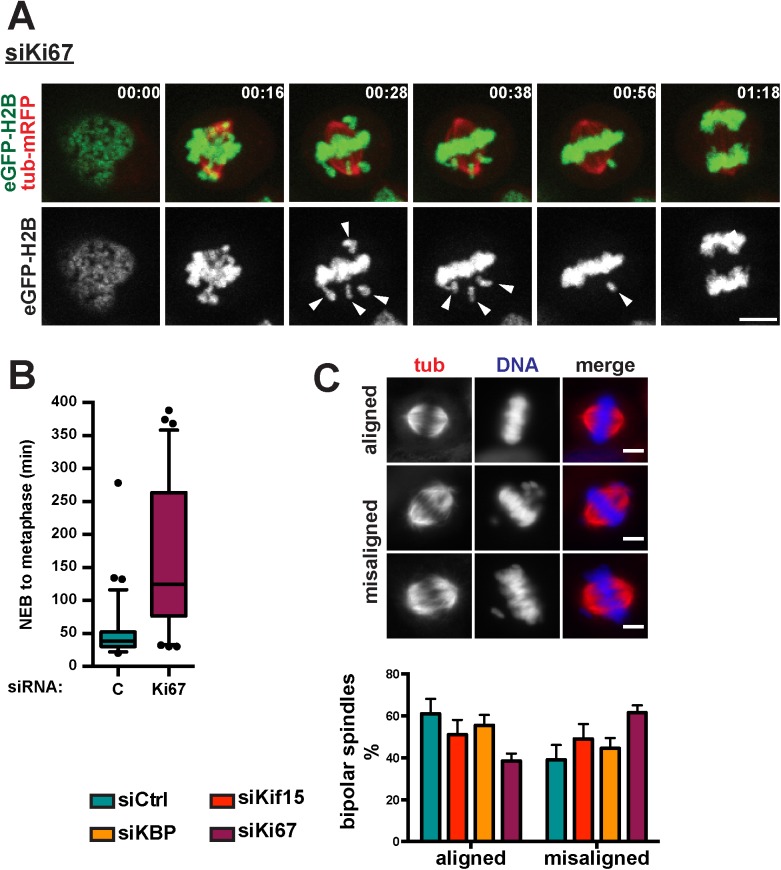
Chromosomally localized Kif15 is required for chromosome alignment. **(A)** Representative stills from the live-cell imaging of Ki67-silenced cells. Arrowheads indicate chromosomes that show less efficient alignment to the metaphase plate. Time is indicated in the upper left corner as hh:mm. Scale bar represents 5μm. **(B)** Quantification of prometaphase duration as observed in the live-cell imaging in control or Ki67-silenced cells. Prometaphase was defined as the time needed for cells to progress from NEBD till anaphase onset. Time points are represented in box-and-whisker plots. Boxes show the upper and lower quartiles (25–75%) with a line at the median, whiskers extend from the 5^th^ to the 95^th^ percentile. Dots represent the outliers (data were compared using Mann-Whitney *U* tests, **** means *p* ≤ 0.0001). **(C)** Upper panel: Representative immunofluorescence images showing bipolar spindles with fully aligned or misaligned chromosomes. Lower panel: Quantification of chromosome alignment in control, KBP, Kif15 or Ki67-silenced cells (scale bar 5, μm). Cells with bipolar spindles were categorized based upon the degree of chromosome alignment, i.e. fully aligned chromosomes (metaphase cells), misaligned chromosomes (most chromosomes have congressed to the metaphase plate, few chromosomes are not aligned yet). Graph shows the average and standard deviation of three independent experiments with at least 61 cells counted per condition, per experiment (data were analyzed using *t* tests, no significant differences were observed).

Our data indicated that KBP is required for Kif15 localization to the spindle equator and chromosome area suggesting that this specific localization of Kif15 may be important for chromosome alignment. Another approach to interfere with this specific localization of Kif15 is through the silencing Ki67 [6]. We therefore followed mitosis by live cell imaging on Ki67-silenced cells [6,10,21] in which Kif15 localization to the chromosomes and spindle equator is completely abolished [6] without interfering with its spindle localization (Fig 4A and 4B). In agreement with the report of Cuylen and colleagues [22], Ki67-silenced cells were significantly delayed in mitosis. Indeed they needed on average 173.9±103.3 min from NEB until the start of cytokinesis instead of 59.6±34.8 min for control cells (*p*<0.0001). The delay was due to an extended prometaphase that lasted 165.5±106.2 min on average instead of 48.6±36.0 min for control cells (*p*<0.0001) (Fig 4A and 4B and S1 and S5 Movies). More than 80% of the Ki67-silenced cells showed chromosome alignment defects. These phenotypes were similar to those of Kif15-and KBP-silenced cells (Figs 3C and 4A). However, Ki67 silencing had additional effects on the organization and dynamics of the MTs within the bipolar spindle. Indeed Ki67-silenced cells often had long and bent spindle MTs and abnormally organized spindle poles with a spiral-like appearance. We verified the results in fixed cells by quantifying the bipolar spindles with all chromosomes aligned versus bipolar spindles with nearly all chromosomes aligned in the different silencing conditions (Fig 4C). Since Kif15 localization to the spindle MTs appears unaffected in Ki67-silenced cells, these results support the idea that the association of Kif15 with the chromosomes at the spindle equator may indeed be required for chromosome alignment [6]. However, the phenotypes observed in the Ki67-silenced cells also indicated that Ki67 has additional functions during mitosis that go beyond its role in Kif15 localization and function.

Taken together, our data suggest that Kif15 and its mitotic partners KBP and Ki67 contribute to chromosome alignment to the metaphase plate. Moreover, our data suggest a mechanism that requires Kif15 localization in the chromosome area, potentially at the MT plus-ends.

### KBP and Kif15 affect K-fiber dynamics

**Fig 5:**
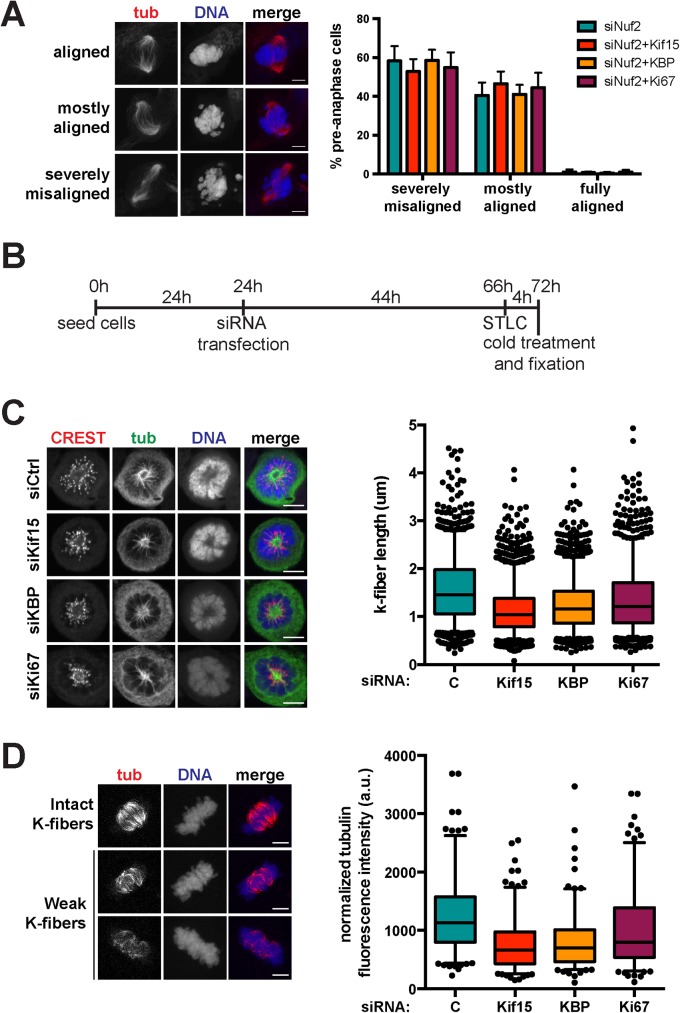
KBP and Kif15 affect K-fiber stability. **(A)** Left panel: Immunofluorescence images of Nuf2-silenced cells showing three different categories of chromosome alignment, i.e. siNuf2 spindles with all chromosomes aligned (fully aligned), with most of the chromosomes aligned (mostly aligned) or with scattered chromosomes (severely misaligned) (scale bar, 5 μm). Right panel: Quantification of the proportion of cells with chromosomes aligned following the specified classification upon in Nuf2 silencing or Nuf2 and Kif15, KBP or Ki67 co-silencing. Graph represents the averages and standard deviations of four independent experiments, counting at least 100 cells per condition, per experiment (data were analyzed using *t* tests, no significant differences were observed). **(B)** Scheme outlining the set-up of the K-fiber measurement experiments. **(C)** Left panel: Immunofluorescence showing representative cells of control, KBP or Kif15-silenced cells treated with STLC and cold before fixation (scale bar, 5 μm). Right panel: Quantification of K-fiber lengths as measured in the immunofluorescence images, represented in box-and-whisker plots. Boxes show the upper and lower quartiles (25–75%) with a line at the median, whiskers extend from the 5^th^ to the 95^th^ percentile. Dots represent the outliers. Data from three independent experiments with at least 20 cells per experiment analyzed (>314 K-fibers measured per condition, per experiment; data were compared using Mann-Whitney *U* tests, **** means *p* ≤ 0.0001). **(D)** Left panel: Immunofluorescence images showing representative cells with intact versus weak K-fibers after 10 min cold treatment before pre-extraction (5 min on ice) and fixation (scale bar, 5 μm). Right panel: Quantification of the normalized tubulin fluorescence intensity in cold-treated spindles upon control, Kif15, KBP or Ki67 silencing. Data are represented in box-and-wisker plots. Boxes show the upper and lower quartiles (25–75%) with a line at the median, whiskers extend from the 5^th^ to the 95^th^ percentile. Dots represent the outliers. Data from three independent experiments with at least 52 cells quantified per condition, per experiment (data were compared using Mann-Whitney *U* tests, **** means *p* ≤ 0.0001).

To better define the role of Kif15 and KBP in chromosome alignment, we then explored their potential role in K-fiber assembly and function. We first examined whether the chromosome alignment defects observed in Kif15, KBP or Ki67-silenced cells required the presence of K-fibers by co-silencing Nuf2 thereby preventing their assembly. Cells in prometaphase and metaphase were classified into three main categories: those with all the chromosomes aligned at the metaphase plate (fully aligned chromosomes), those with a majority of chromosomes at the metaphase plate but a few away from it (few misaligned chromosomes) and those with severely misaligned chromosomes (scattered chromosomes). As shown in Fig 5A, similar chromosome misalignment defects were observed in the Nuf2-silenced cells and in cells co-silenced for Nuf2 and either Kif15, KBP or Ki67. These results suggest that the role of Kif15 and its mitotic partners KBP and Ki67 in chromosome alignment is most probably related to K-fiber assembly and/or dynamics.

Since Kif15 associates with the K-fibers (K-MTs) [6,11,14], we then explored whether it may play a role in their assembly by measuring the length of the K-fibers present in control and Kif15, KBP or Ki67-silenced cells. To facilitate the quantification, cells were first incubated in STLC to promote the formation of monopolar spindles. The cells were then placed on ice, fixed and processed for immunofluorescence as previously described [23] (Fig 5B). In all three silencing conditions K-fibers were 20–30% shorter than in control cells, indicating that Kif15 and its partners KBP and Ki67 are important for K-fiber assembly and/or stability (Fig 5C). We then examined K-fiber stability in the silenced cells. Cells were placed on ice for 10 min to promote the depolymerization of the less stable spindle MTs while maintaining the bundled K-fiber MTs. We then quantified K-fiber stability by measuring the total tubulin fluorescence intensity in pre-extracted fixed cells and processed for immunofluorescence. We found that K-fiber MTs were reduced by 20–40% in cells silenced for Kif15, KBP or Ki67 compared to controls (Fig 5D). Altogether these results suggested that Kif15 and its mitotic partners KBP and Ki67 are required for the assembly of stable K-fibers of the correct length.

## Discussion

Kif15 has been shown to play a role in bipolar spindle formation in concert with the dominant bipolar kinesin Eg5, providing pushing forces that separate the centrosomes/spindle poles after NEBD [6,7,10]. Here we identified KBP as a mitosis specific interaction partner of Kif15 and describe for the first time its distribution and function during mitosis. KBP has been previously shown to interact with various kinesins [17,24,25]. It has been recently proposed to function as an inhibitor of a subset of kinesins promoting their dissociation from the MTs thereby contributing to the regulation of MT dynamics and cargo transport in neuronal cells [20].

Here we found that KBP interacts with Kif15 specifically and exclusively during mitosis and that both are required for the efficient alignment of all the chromosomes to the metaphase plate. Indeed, in the absence of any of these proteins or both, cells are delayed in prometaphase with a small number of misaligned chromosomes. Similar defects occur in cells silenced for Ki67, another Kif15 partner. Interestingly both KBP and Ki67 [6] are required for Kif15 localization to the spindle equator and chromosomal area without apparently interfering with its spindle localization. Other groups have reported Kif15 localization to specific locations. Sturgill and colleagues reported that Kif15 co-localizes with Hec1, a component of the essential kinetochore-associated NDC80 complex [10]. Moreover, Kif15 was recently shown to accumulate at MT plus-ends *in vitro* [14]. Altogether these data strongly suggest that Kif15 localizes to or accumulates at the spindle microtubule plus-ends in the proximity of the chromosomes and that this localization may be regulated positively by its associated partners KBP and Ki67. Our results suggest that this localization of Kif15 is required for the bi-orientation or alignment of chromosomes since all the experimental conditions that abolish (silencing of Kif15 or Ki67) or reduce (KBP silencing) this pool of Kif15 result in a delay in chromosome alignment.

The mechanism by which Kif15 contributes to chromosome alignment does not seem to involve polar ejection forces (PEFs) [26–28]. Indeed, Kif15 silencing does not interfere with chromosome arms positioning away from the center of STLC-induced monopolar spindles (Fig 5C) [26], suggesting that PEFs are active in these cells. Since we did not observe stronger chromosome alignment defects in cells co-silenced for Kif15 and Nuf2 (like those reported in CENP-E and Nuf2 co-silenced cells [29]) (Fig 5A), our data rather point to a function of Kif15 and its associated partners KBP and Ki67 at the level of the K-fibers. Indeed, the three proteins are required for K-fiber stability. Interestingly, Kif15 was shown to bundle MTs *in vitro*, and to associate with K-fibers in cells [6,10,11]. The mechanism involved is however still unclear. It was recently described that Kif15 has two MT-binding sites, one ATP-dependent site in the motor head, and another ATP-independent site in the first coiled-coil domain in the stalk region [11]. This could provide a MT crosslinking activity important for K-fiber assembly and stability. However, other authors found that Kif15 can form homotetramers [14] that can crosslink MTs and move them relative to one another, promoting the formation of parallel microtubule arrays. These properties could explain particularly well the role of Kif15 in K-fiber assembly *in vivo*. The defects in K-fiber assembly may also result in defects in chromosome attachment that would explain the misalignment phenotype as well as the delay in mitosis.

The specific role of KBP as a partner of Kif15 in chromosome alignment is still unclear. Using MT pelleting and kinesin motility assays Kevenaar et al (2016) recently reported that KBP binds to the motor domain of the neuronal motor Kif1A and negatively regulates its binding to MTs. Our data do not indicate a major displacement of Kif15 from the spindle MTs in the absence of KBP. Instead our data show that it is required for Kif15 localization to the spindle equator and chromosome area. Therefore the regulation of Kif15 motility and/or MT binding properties by KBP in mitosis may be more complex than a simple inhibition of MT-binding though its motor domain. Other possibilities will have to be tested. In particular, it will be interesting to determine whether KBP may modify the motility and MT plus-end binding properties of Kif15 *in vitro*.

Kevenaar et al (2016) found that KBP interacts with a number of kinesins and in particular mitotic motors like Kif15 but also Kif18a. Since Kif18a has been shown to play a role in chromosome alignment [30] it will therefore be important to determine whether KBP also interacts with Kif18a during mitosis and whether this interaction may play a role in its function.

In summary, our work has established a role for Kif15 in chromosome alignment. This function is associated to the localization of Kif15 to the chromosomal area potentially its accumulation at the MT plus-ends, a localization that is regulated by Ki67 and a novel mitotic Kif15 partner KBP. Further work will be needed to understand the mechanism by which these proteins ensure that all the chromosomes become aligned to the metaphase plate.

## Materials and methods

### Plasmid construction, protein production and antibodies

The full-length KIF15 cDNA (residues 1–1388) was cloned into pFlag-CMV2 as previously described [6]. The construct pEGFP-N1-hKBP was a generous gift from Maria Alves (University of Groningen, The Netherlands). To generate 3xFlag-tagged KBP, the full-length cDNA was amplified from pEGFP-N1-hKBP using 5’- TCTAGCGTTTAAACTTAACCATGGACTACAAAGACCATGA-CGGTGATTATAAAGATCATGATATCGATTACAAGGATGACGATGACAAGATGGCGAACGTTCCGTGG-3’ and 5’-GGTTTAAACGGGCCCTCTAGACTTA-AGTCAGGGCCATCTTGGTTCTGAATCT-3’ and cloned into the pcDNA5/FRT/TO vector (Invitrogen) using the Gibson assembly method [31]. For rescue experiments, 4 silent mutations were introduced in the seed region of the KBP siRNA by two consecutive rounds of site directed mutagenesis using 5’-CAGATCCAGTTTGAAATTGCACAcGCgTACTATGATATGATGG-3’, 5’-CCATCATATCATAGTAcGCgTGTGCAATTTCAAACTGGATCTG-3’, 5’-CC- AGTTTGAAATTGCACAcGCgTAtTAcGATATGATGGATTTGAAGG-3’ and 5’-CCTtCAAATCCATCATATCgTAaTAcGCgTGTGCAATTTCAAACTGG-3’.

For expression of the recombinant Kif15 motor domain, the first 714 amino acids of human Kif15 were amplified from the Flag-Kif15 expression construct [6] using 5’-TATACATATGGCTAGCATGGCACCCGGCTGCAAA-3’ and 5’-GGTGGTGGTGCTCGAGGGCCTCAAAAGCTTGTTCATTCATCTC-3’, and cloned into pET-21b (Novagen) using Gibson assembly. Kif15-M-His protein was produced in BL21 (DE3) E. coli cells and purified using Ni-NTA resin (Qiagen) following manufacturers instructions, after which the protein was concentrated and the buffer was changed to motor buffer (100mM PIPES, 2mM MgAc_2_, 1mM EGTA, 1mM DTT) using Amicon Ultra centrifugal filter units (Millipore).

For expression of recombinant His-tagged KBP, the full-length cDNA was amplified from pEGFP-N1-hKBP using 5’-GTCGGGATCCGAATTCTATGGCGA-ACGTTCCGTGG-3’ and 5’-GGTGGTGGTGCTCGAGAGTCAGGGCCATCTTG-GTT-3’ and cloned into pET-21b using Gibson assembly. KBP-His protein was produced in BL21 (DE3) E. coli cells and purified using Ni-NTA resin (Qiagen) following manufacturers instructions, after which the protein was concentrated and the buffer was changed to PBS using Amicon Ultra centrifugal filter units (Millipore). Purified KBP-His protein was used for immunizing rabbits to generate the anti-KBP antibody following a standard three weeks protocol at the antibody facility of the University of Barcelona. Sera were affinity purified against KBP-His protein covalently bound to a HiTrap column (GE Healthcare) following manufacturers instructions. Affinity purified anti-KBP was used at 5μg/ml in immunofluorescence and 1μg/ml in immunoblotting.

The rabbit polyclonal antibody against Kif15 was previously described [6], and was used at 5μg/ml in immunofluorescence and 0.43μg/ml in immunoblotting. The following antibodies were used: anti-α-tubulin (DM1A; Sigma) at 1:10000 for immunoblotting and 1:1000 in immunofluorescence; polyclonal anti-**β**-tubulin (Abcam) was used at 1:200 in immunofluorescence; polyclonal anti-Hec1 (GeneTex) at 1:100 in immunofluorescence; human anti-kinetochores (CREST, Antibodies Incorporated) at 1:100 in immunofluorescence; mouse anti-Flag (Sigma) was used at 1:200 in immunofluorescence and 1:1000 in immunoblotting; anti-Ki67 (BD Bioscience) was used at 1:250 in immunoblotting; monoclonal anti-His (Sigma) was used at 1:1000 in immunoblotting. Secondary antibodies were anti-rabbit, anti-mouse or anti-human antibodies conjugated to Alexa-488, 568, 680 (Molecular Probes) or IRDye800 (Licor) were used at 1:1000 in immunofluorescence and 1:10000 in immunoblotting.

### Cell culture and siRNA/plasmid transfections

Both HEK293T and HeLa cells were grown at 37°C in DMEM (Lonza) containing 10% fetal bovine serum (Invitrogen), 2mM L-glutamine (Invitrogen) and 100U/ml penicillin (Invitrogen) and 100μg/ml streptomycin (Invitrogen) with 5% CO_2_ in a humid atmosphere. HeLa cells stably expressing H2B–eGFP/**α**-tubulin–mRFP were a gift from P. Meraldi (ETH, Zurich). These cells were cultivated in medium as above, supplemented with 600μg/ml G418 (Sigma) and 2.5μg/ml puromycin (Sigma). Medium of HEK293T cells stably expressing Flag-tagged Kif15 or KBP was supplemented with 2.5μg/ml puromycin.

Silencing of luciferase (as a control), Kif15 and Ki67 were performed using siRNA oligos as previously described [6]. KBP silencing was performed using the SMARTpool or the single siRNA (5’-GCACATGCTTACTATGATA-3’) obtained from Dharmacon. siRNAs for Nuf2 (sequence from [32]) were obtained from Dharmacon. Transfections were performed using 100pmol of siRNA per well of a 6-well plate and the Lipofectamine RNAiMAX reagent (Invitrogen) according to the manufacturers instructions. Double silencing experiments were performed with a total amount of 100pmol siRNA in either an 8:2 ratio (for combinations with Kif15) or a 5:5 ratio (for all other combinations).

Plasmid transfections of HeLa cells were performed using the XtremeGene9 DNA transfection reagent (Roche) according to manufacturers instructions. Plasmid transfections of HEK293T cells were performed using calcium phosphate precipitation and subsequent glycerol shock as described [33]. HEK293T cells stably expressing Flag-tagged Kif15 or KBP were generated after co-transfection with the pBABE-puro plasmid [34] (in a 9:1 ratio). 24 hours after transfection cells were diluted, and selected in puromycin (2.5μg/ml) containing medium 24 hours later. Cells were kept under selection until single colonies could be picked and expanded. Cells arising from single colonies were tested for presence of the transgene by immunoblotting.

To determine the cell cycle regulated expression of KBP, cells were synchronized by double thymidine block. In short cells were blocked during 24h in medium supplemented with 4mM thymidine, then released in normal medium for 16h and blocked again during 24h in medium with 4mM thymidine. After the second block cells were collected every 2h after release. KBP levels were determined by gel electrophoresis and immunoblotting.

### Live cell imaging

Cells were seeded in 15mm glass bottom LabTek dishes and transfected with the siRNA 24 hours later. The morning after transfection, cells were synchronized using a single thymidine block, i.e. cells were incubated with 2mM thymidine (Sigma) for 18h, then released into imaging medium (complete medium without phenol red, Lonza) after three washes with PBS. Approximately 9 hours after thymidine release cells were imaged on an Olympus IX81 Andor Revolution XD spinning disk microscope equipped with an incubator chamber. Eight optical sections (1μm z-stack intervals) were acquired every 2 min for a total time of 5 hours using a Plan S Apo 60x oil immersion objective (NA 1.45). Movies were processed and analyzed using ImageJ.

### Immunofluorescence

Cells were grown on coverslips and fixed for 10 minutes at -20°C cold methanol (Merck). For staining with the anti-KBP antibody cells were pre-extracted for 5 sec in pre-extraction buffer (1xPHEM (60mM PIPES (Sigma), 25mM Hepes (Sigma), 10mM EGTA (Sigma), 2mM MgCl2 (Sigma), pH 6.9), 0.5% Triton X-100 (Sigma), 1mM DSP (Sigma)), fixed in 4% paraformaldehyde (Sigma) in 1xPHEM for 15 min at 37°C and quenched in 100mM glycine in PBS for 5 min at 37°C. After blocking in PBS; 2% BSA (Sigma); 0.1% Triton X-100 for 20 min, the coverslips were incubated with primary antibodies in PBS; 2% BSA; 0.1% Triton X-100 for 1 hour at room temperature. Coverslips were washed 3 times 10 minutes in PBS, 0.1% Triton X-100. Fluorescently labelled secondary antibodies (Alexa-488 or Alexa-568 secondary antibodies, Sigma) along with the DNA staining dye Hoechst 33342 (Invitrogen) were incubated as described above for 45 minutes. The coverslips were mounted on Mowiol (0.1 M Tris-HCl (Sigma) pH 8.5; 25% Glycerol (Merck); 10% Mowiol 4–88 (Calbiochem); 2.5% DABCO (1,4-diazobicyclo-[2.2.2]-octane; Merck).

Fixed cells were visualized with a ×63 objective on an inverted DMI-6000 Leica wide-field fluorescent microscope. Three-dimensional images were acquired in 0.5 μm steps using a ×63 oil-immersion (NA 1.3) objective lens on a Leica SPE confocal microscope. Pictures were acquired using the Leica Application Suite software. The optical section images were projected on a single plane. Images were processed with ImageJ and mounted in figures using Illustrator (Adobe).

### Immunoprecipitations

For immunoprecipitation and Flag pull-downs HEK293T cells were plated on 0.1% Poly-D-Lysin coated flasks and synchronized using a single thymidine block (2mM) overnight, released for 6 hours and blocked using nocodazole (2μM) overnight. The next morning cells were released from the nocodazole block and approximately one hour later mitotic cells were collected by mitotic shake off. Cell pellets were resuspended in extraction buffer (20mM Hepes pH7.8, 175mM NaCl, 2.5mM MgCl_2_, 10% glycerol, 1mM DTT (Sigma), complete EDTA-free protease inhibitors (Roche), 20mM **β**-glycerolphophate (Sigma), 5mM sodium fluoride (Sigma), 100μM sodium orthovanadate) and lysed using nitrogen decompression by applying 1500–2000 PSI pressure for 15 minutes at 4°C. Lysates were centrifuged for 15 minutes and the supernatants were incubated with antibody-coated protein-A dynabeads (Invitrogen) or with anti-Flag M2 magnetic beads (Sigma) at 4°C for 2h or 30 min, respectively. After several washes, the immunoprecipitated proteins were eluted from the beads with SDS sample buffer or with Flag peptide (Sigma) and analyzed by western blot.

To assess the phosphorylation dependency of the Kif15-KBP interaction, Flag-Kif15 pull-downs were treated with **λ**-phosphatase on beads in the absence of phosphatase inhibitors. In short, pull-downs were performed as described above and after washing, the beads were incubated with 400U **λ**-phosphatase (New England Biolabs) according to manufacturers instructions for 20 minutes at 30°C. After this incubation the beads were washed and the proteins were eluted using Flag peptide.

### SILAC proteomics

HEK293T and stably overexpressing Flag-tagged Kif15 HEK293T cells were cultivated and expanded in respectively light media (DMEM-14, Dundee cell products) or media containing the heavy isotopes of arginine and lysine (DMEM-17, Dundee cell products) supplemented with 10% SILAC dialyzed FCS (D-FCS100, Dundee cell products) over a period of 10 days prior to synchronization. Then cells were plated on 0.1% Poly-D-Lysin coated flasks and synchronized with a single thymidine block (2mM) overnight, released for 8 hours and blocked using nocodazole (2 μM) overnight. The next morning cells were released from the nocodazole block and approximately one hour later mitotic cells were collected by mitotic shake off. Cell pellets were resuspended in extraction buffer (20mM Hepes pH7.8, 175mM NaCl, 2.5mM MgCl_2_, 10% glycerol, 1mM DTT (Sigma), complete EDTA-free protease inhibitors (Roche), 800μM PMSF, 20mM **β**-glycerolphophate (Sigma), 5mM sodium fluoride (Sigma), 100μM sodium orthovanadate) and lysed using nitrogen decompression by applying 1500–2000 PSI pressure for 15 minutes at 4C. Protein concentrations were determined using Bradford reagent (Bio-Rad) and equal amounts of protein of HEK293T and Flag-Kif15 HEK293T lysates were combined for subsequent Flag pull-downs. Flag pull-downs were performed using the 20μl of Anti-Flag M2 affinity gel (Sigma) per mg protein lysate. Beads were incubated protein lysates for 15 minutes at 4C, after which 1 wash step using wash buffer 1 (20mM Tris pH 7.9, 10% glycerol, 0.2mM EDTA, 10mM **β**-mercaptoethanol, 500mM KCl, 0.1% NP-40 supplemented with complete EDTA-free protease inhibitors (Roche), 800 μM PMSF, 20mM **β**-glycerolphophate (Sigma), 5mM sodium fluoride (Sigma), 100 μM sodium orthovanadate) and 1 wash step using wash buffer 2 (20mM Tris pH 7.9, 0.2mM EDTA, 10mM **β**-mercaptoethanol, 50mM KCl supplemented with complete EDTA-free protease inhibitors (Roche), 800 μM PMSF, 20mM **β**-glycerolphophate (Sigma), 5mM sodium fluoride (Sigma), 100 μM sodium orthovanadate). Proteins were eluted from the beads using Flag peptide (Sigma). The eluted sample was concentrated by speedvac centrifugation and proteins were resolved by polyacrylamide gel electrophoresis. Proteins were visualized by colloidal blue staining of the gel (Novex, Invitrogen) after which bands were excised and trypsinized. Briefly: gel bands were destained with 40% ACN/100mM ABC, samples were reduced with dithiothreitol (2μM, 30min, 56°C) and alkylated in the dark with iodoacetamide (10 μM, 30 min, 25°C). Gel bands were then dehydrated with ACN and digested with 0.3 μg of trypsin (Promega) overnight at 37°C. After digestion, peptides were extracted and cleaned up on a homemade Empore C18 column (3M) [35]. After digestion, samples were analyzed using an LTQ-Orbitrap XL mass spectrometer (Thermo Fisher Scientific) coupled to an Agilent Technologies 1200 Series (Agilent Technologies). Peptides were loaded onto C18 Zorbax precolumn (Agilent Technologies) and were separated by reversed-phase chromatography using a 12-cm column with an inner diameter of 75 μm, packed with 5 μm C18 particles (Nikkyo Technos Co.). Chromatographic gradients started at 97% buffer A and 3% buffer B with a flow rate of 300nl/min, and gradually increased to 90% buffer A and 10% buffer B in 1 min, and to 65% buffer A / 35% buffer B in 60 min. After each analysis, precolumn and column were washed for 10 min with 10% buffer A / 90% buffer B. Buffer A: 0.1% formic acid in water. Buffer B: 0.1% formic acid in Acetonitrile. The mass spectrometer was operated in positive ionization mode with nanospray voltage set at 2.5 kV and source temperature at 200°C. Ultramark 1621 for the FT mass analyzer was used for external calibration prior the analyses. Moreover, an internal calibration was also performed using the background polysiloxane ion signal at m/z 445.1200. The instrument was operated in DDA mode and full MS scans with 1 micro scans at resolution of 60.000 were used over a mass range of m/z 350–2000 with detection in the Orbitrap. Auto gain control (AGC) was set to 1E6, dynamic exclusion (60 seconds) and charge state filtering disqualifying singly charged peptides was activated. In each cycle of DDA analysis, following each survey scan the top ten most intense ions with multiple charged ions above a threshold ion count of 5000 were selected for fragmentation at normalized collision energy of 35%. Fragment ion spectra produced via collision-induced dissociation (CID) were acquired in the Ion Trap, AGC was set to 5e4, isolation window of 2.0 m/z, activation time of 0.1ms and maximum injection time of 100 ms was used. All data were acquired with Xcalibur software v2.2.

The MaxQuant software suite (v1.3.0.5) was used for peptide identification and quantification [36]. The data was searched against an in-house generated database containing all proteins corresponding to *Homo Sapiens* in the Swissprot database, a list of common contaminants and the corresponding decoy entries (release February 2012, 37366 entries). A precursor ion mass tolerance of 4.5 ppm at the MS1 level was used, and up to three missed cleavages for trypsin were allowed. The fragment ion mass tolerance was set to 0.5 Da. Oxidation of methionine, protein acetylation at the N-terminal, defined as variable modification; whereas carbamidomethylation on cysteines was set as a fix modification. A multiplicity of 2 was used, and Arg10 and Lys8 heavy labels were selected. Peptides have been filtered respectively using a 1%FDR and peptide areas were used to calculate heavy-to-light ratios.

### Cold-stable assays

For measuring tubulin intensities in cold-treated spindles cells grown on coverslips were washed with PBS and incubated on ice for 10 minutes in L15 medium (Sigma) supplemented with 20mM HEPES at pH 7.3. Cells were pre-extracted (1xPHEM, 0.5% Triton X-100 (Sigma)) for 5 min on ice, and fixed in 4% paraformaldehyde (Sigma) in 1xPHEM for 15 min at room temperature and quenched in 100mM glycine in PBS for 15 min at room temperature. Coverslips were processed for immunofluorescence to visualize MTs and DNA. To quantify tubulin intensities we used a modified version of the method described in [37]. Images were processed with ImageJ: a circle was drawn encompassing the whole spindle, tubulin fluorescence and the area were measured in this circle (I_small_, A_small_). Then a concentric circle of twice the area of the spindle circle was used to measure the background fluorescence (I_bg_ = I_large_-I_small_). Fluorescence intensities attributed to the spindle (I_spindle_ = I_small_-I_bg_) were then normalized against the average background fluorescence. Data was obtained from three independent experiments (n ≥ 52 cells, each). For K-fiber length measurements, cells were incubated in 10μM STLC prior to cold-treatment. After 5 hours of STLC treatment cells were washed once with PBS and incubated on ice for 6.5 min in L15 medium (Sigma) supplemented with 20mM HEPES at pH 7.3. Then cells were fixed 10 min in MeOH at -20°C and processed for immunofluorescence to visualize MTs, CREST and DNA (two independent experiments, n ≥ 30 cells, each).

### Generation of Eg5 independent cells (EICs)

HeLa cells were cultivated in medium containing increasing amounts of STLC until a final concentration of 40 μM STLC was reached an individual clones could be isolated and clonally expanded. To assess the Eg5 independency, Eg5 was silenced by siRNA treatment, and the proportion of bipolar spindles was determined.

### In vitro interaction

The in vitro interaction potential of Kif15-M-His and KBP-His proteins was assessed using gel filtration chromatography and sucrose gradient centrifugation. For gel filtration chromatography 200 μl samples of either 80μg Kif15-M-His or KBP-His or both in motor buffer (100mM PIPES, 2mM MgAc2, 1mM EGTA, 1mM DTT) were pre-incubated for 30 minutes on ice and subsequently loaded onto a Superose 6 column (GE Healthcare). For sucrose gradient centrifugation, 200μl of 80μg Kif15-M-His or KBP-His or both in motor buffer were pre-incubated for 30 minutes on ice and subsequently loaded onto 4 ml 6–28% sucrose gradients in motor buffer and spun for 16 h at 4°C at 26000 rpm in a SW60 rotor. Samples were collected in 200μl fractions after centrifugation. The profile of Kif15-M-His and KBP-His in both cases was determined by gel electrophoresis and immunoblotting with the anti-His (detecting both recombinant proteins) and anti-KBP antibodies. To obtain the sedimentation coefficient of the proteins, the following standards were run in parallel in sucrose gradients under the same conditions: ovalbumin, aldolase and thyroglobulin (Gel Filtration Calibration Kit HMW from GE Healthcare) and catalase and BSA (from Sigma). To obtain the Stokes radius of the proteins the following protein standards were used in gel filtration under the same conditions: aldolase, thyroglobulin and dextran blue (Gel Filtration Calibration Kit HMW from GE Healthcare) and BSA and catalase (Sigma). The native molecular weights of each individual protein and of the Kif15-KBP complex were calculated using the values obtained for the Stokes radius and the sedimentation coefficients, as described in [38].

### Statistical analysis

Statistically significant differences between the experimental data were assessed using GraphPad Prism 6 software (GraphPad Software, La Jolla, CA) using non-parametric two sample tests (Mann-Whitney *U* tests, in case the data sets were not normally distributed) or unpaired two-sample *t* tests (for normally distributed data sets).

## Supporting information

S1 Fig **(A)** Summary of SILAC data identifying KBP as a novel Kif15 interactor in three independent experiments. **(B)** Values for the Stokes radius and sedimentation coefficients of the recombinant Kif15-M-His and KBP-His proteins individually and in combination (with or without ATP) obtained from the gel filtration and sucrose gradient experiments. The calculated native molecular weights are shown.
(TIF)

S2 Fig **(A)** Representative immunofluorescence images showing Kif15 localization at different stages of mitosis in control versus KBP-silenced cells (scale bar, 5 μm). **(B)** Quantification of the spindle length in control and KBP-silenced cells (average of at least 42 cells quantified per condition in two independent experiments; data were compared using an unpaired *t* test, no significant differences were observed). **(C)** HeLa and HeLa EICs were control, Eg5, KBP or Kif15-silenced and the number of bipolar versus monopolar structures were scored. Bars represent the proportion of monopolar spindles (averages with standard deviation of four independent experiments with at least 35 cells counted per condition, per experiment; data were compared using unpaired *t* tests, **** means *p* ≤ 0.0001). **(D)** Quantification of chromosome alignment in control and KBP-silenced cells either transiently expressing empty vector or siRNA-resistant flag-tagged KBP. Cells with bipolar spindles were categorized based upon the degree of chromosome alignment, i.e. fully aligned chromosomes (metaphase cells), misaligned chromosomes (most chromosomes have congressed to the metaphase plate, few chromosomes are not aligned yet). Graph shows the average and standard deviation of three independent experiments with at least 48 cells counted per condition, per experiment (data were compared using unpaired *t* tests, *** means *p* ≤ 0.001). **(E)** Representative immunofluorescence image showing anti-Flag staining in a metaphase HeLa cell overexpressing 3xFlag-tagged KBP (scale bar 5 μm).
(AI) Click here for additional data file.

S3 Fig **(A)** Quantification of mitosis duration as observed in the live-cell imaging in control and Kif15-, KBP-, or Kif15/KBP-silenced cells. The duration of mitosis was defined as the time needed for cells to progress from NEBD till start of telophase (determined as the time of contraction of the cleavage furrow). Time points are represented in box-and-whisker plots. Boxes show the upper and lower quartiles (25–75%) with a line at the median, whiskers extend from the 5^th^ to the 95^th^ percentile. Dots represent the outliers (data were compared using Mann-Whitney *U* tests, ** means *p* ≤ 0.01, **** means *p* ≤ 0.0001).
(AI) Click here for additional data file.

S1 Movie Representative movie from the live-cell imaging of control-silenced cells that constitutively express H2B-GFP (green) to visualize the chromosomes and Tub-RFP (red) to visualize microtubules. Time is indicated in the upper left corner as hh:mm.
(AVI)

S2 Movie Representative movie from the live-cell imaging of KBP-silenced cells that constitutively express H2B-GFP (green) to visualize the chromosomes and Tub-RFP (red) to visualize microtubules. Time is indicated in the upper left corner as hh:mm.
(AVI)

S3 Movie Representative movie from the live-cell imaging of Kif15-silenced cells that constitutively express H2B-GFP (green) to visualize the chromosomes and Tub-RFP (red) to visualize microtubules. Time is indicated in the upper left corner as hh:mm.
(AVI)

S4 Movie Representative movie from the live-cell imaging of KBP- and Kif15-silenced cells that constitutively express H2B-GFP (green) to visualize the chromosomes and Tub-RFP (red) to visualize microtubules. Time is indicated in the upper left corner as hh:mm.
(AVI)

S5 Movie Representative movie from the live-cell imaging of Ki67-silenced cells that constitutively express H2B-GFP (green) to visualize the chromosomes and Tub-RFP (red) to visualize microtubules. Time is indicated in the upper left corner as hh:mm.
(AVI)
