# Supplementary figures and images for: Role of Kif15 and its novel mitotic partner KBP in K-fiber dynamics and chromosome alignment
Source: PLoS One. 2017 Apr 26;12(4):e0174819. doi: 10.1371/journal.pone.0174819 (PMC5405935; doi:10.1371/journal.pone.0174819)

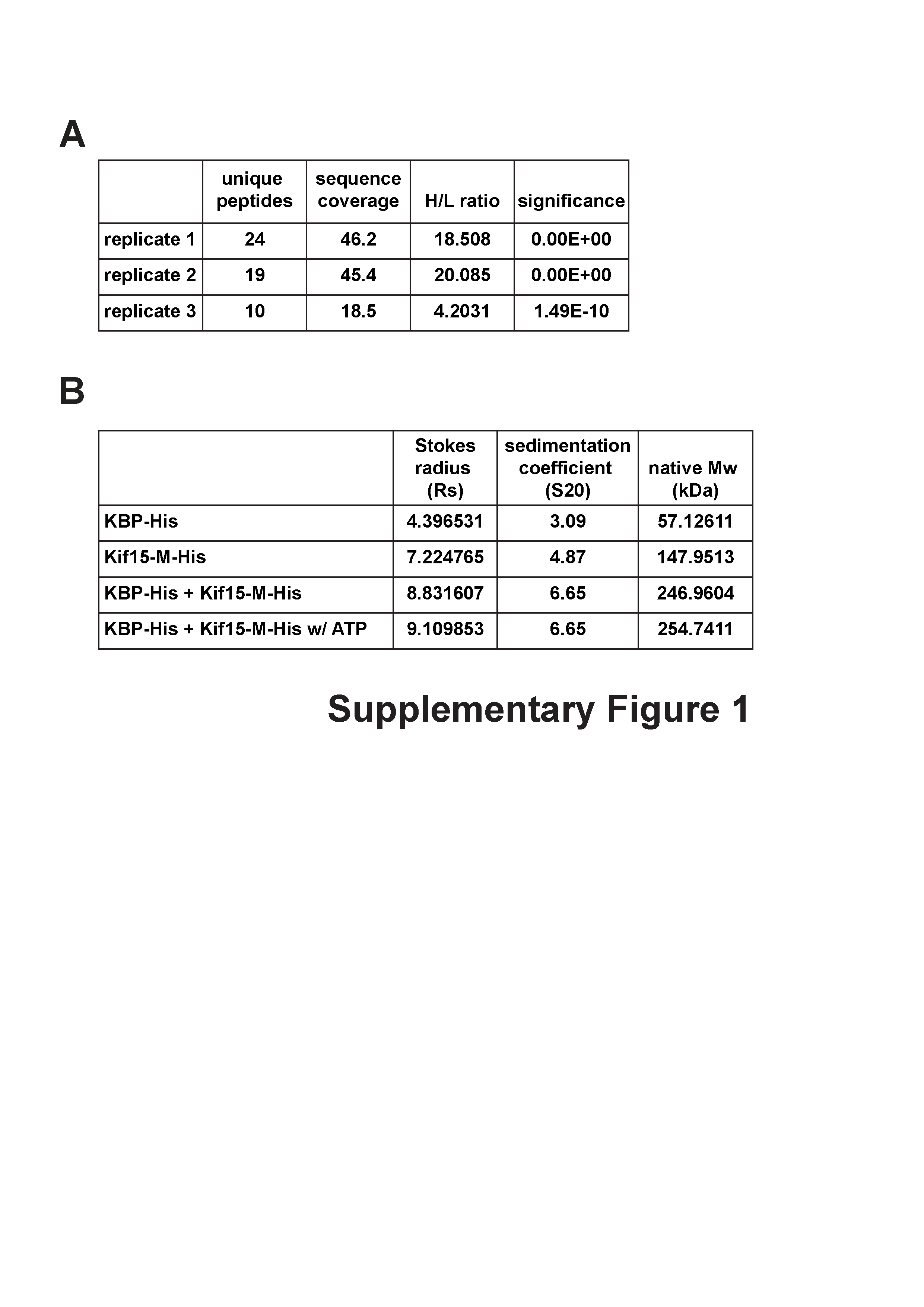

Supplement: S1 Fig — (A) Summary of SILAC data identifying KBP as a novel Kif15 interactor in three independent experiments. (B) Values for the Stokes radius and sedimentation coefficients of the recombinant Kif15-M-His and KBP-His proteins individually and in combination (with or without ATP) obtained from the gel filtration and sucrose gradient experiments. The calculated native molecular weights are shown. (TIF) [file pone.0174819.s001.tif]
